# Angiotensin II Blood Serum Levels in Piglets, after Intra-Dermal or Intra-Muscular Vaccination against PRRSV

**DOI:** 10.3390/vetsci9090496

**Published:** 2022-09-11

**Authors:** Georgios Maragkakis, Labrini V. Athanasiou, Laskarina-Maria Korou, Serafeim C. Chaintoutis, Chrysostomos Dovas, Despina N. Perrea, Georgios Papakonstantinou, Georgios Christodoulopoulos, Dominiek Maes, Vasileios G. Papatsiros

**Affiliations:** 1 Clinic of Medicine, Faculty of Veterinary Medicine, University of Thessaly, 43100 Karditsa, Greece; 2 Laboratory for Experimental Surgery and Surgical Research, Medical School, National and Kapodistrian University of Athens, 11527 Athens, Greece; 3 Diagnostic Laboratory, School of Veterinary Medicine, Faculty of Health Sciences, Aristotle University of Thessaloniki, 54627 Thessaloniki, Greece; 4 Department of Internal Medicine, Reproduction and Population Medicine, Faculty of Veterinary Medicine, Ghent University, 9820 Merelbeke, Belgium

**Keywords:** pig, porcine reproductive and respiratory syndrome, intradermal, vaccine, Ang II

## Abstract

**Simple Summary:**

Porcine reproductive and respiratory syndrome virus (PRRSV) infection causes massive financial losses in pig production worldwide. Vaccination is still the most cost-effective tool to handle PRRSV infection. PRRSV induces apoptosis in different organs. Angiotensin II (Ang II) participates in the inflammatory response, cell proliferation, migration, and apoptosis. The objective of the current study was to assess the concentration of Ang II in the serum of piglets following immunization against PRRSV through intradermal (ID) or intramuscular (IM) vaccination with a commercial PRRS modified live virus (MLV) vaccine. The results indicated differences in viremia of tested piglets at 7 weeks of age, while piglets at 10 weeks of age were all found qRT-PCR positive for PRRSV. Moreover, significant differences were noticed in Ang II in 7-week-old piglets. In conclusion, our study provides evidence that ID vaccination induces less tissue damage, based on the lower measurements of Ang II in the serum of ID vaccinated piglets.

**Abstract:**

The Porcine Reproductive and Respiratory Syndrome Virus (PRRSV) induces apoptosis in different organs. Angiotensin II (Ang II) is the main effector of the renin-angiotensin system and participates in apoptosis. Thus, this study aimed to investigate changes in piglet serum Ang II levels following intradermal (ID) and intramuscular (IM) vaccination with a commercial PRRS modified live virus (MLV) vaccine. The trial was conducted in a commercial pig farm, including 104 piglets which were randomly allocated to four groups: Group A—Porcilis PRRS ID, Group B—Porcilis PRRS IM, Group C—Diluvac ID and Group D—Diluvac IM. The study piglets were either vaccinated or injected at 2 weeks of age and they were tested by qRT-PCR for PRRSV and by ELISA for Ang II. The results indicated differences in viremia of tested piglets at 7 weeks of age, while piglets at 10 weeks of age were all found qRT-PCR positive for PRRSV. In addition, significant differences were noticed in Ang II in 7-week-old piglets. In conclusion, the present study provides evidence that ID vaccination induces less tissue damage, based on the lower measurements of Ang II in the serum of ID vaccinated piglets.

## 1. Introduction

Porcine reproductive and respiratory syndrome virus (PRRSV) infection has a significant economic impact on pig production globally [1], due to reproductive failure in sows and respiratory disease in piglets and fattening pigs [2]. Vaccination remains the most crucial and cost-effective preventive tool in the fight to limit PRRS infection and its consequences [3,4]. Commercial PRRSV modified live virus (MLV) or killed virus (KV) vaccines are now available for use in sows or piglets and sows, in many countries worldwide. However, modern available vaccines do not offer effective prevention of viruses spreading within a herd [1,3]. Experimental challenge and field studies reported that PRRSV-MLVs create delayed but effective protection against wild-type PRRSV strains with genetic homology, while they provide only partial or no protection at all against heterologous strains [1,5,6]. 

Intramuscular (IM) vaccination, using needles, has usually been applied on pig farms; however, risks associated with needles have increased. Intradermal (ID) vaccination has the advantage, compared with IM administration, of easy access to dendritic cells at the site of injection, and targeting antigen-presenting cells in the epidermis near skin-draining lymph nodes [7,8]. This route of vaccine administration induces a faster and more direct response of the vaccinated pigs to the antigen-induced with the vaccine. ID vaccination in pigs has been reported to provide efficient protection of the pigs against infections with other pathogens, such as Aujeszky’s disease [9], PRRSV [10,11,12,13,14], Porcine Circovirus 2 (PCV2) [15,16] and porcine enzootic pneumonia (*Mycoplasma hyopneumoniae*) [17,18]. ID vaccination is proposed as an alternative vaccination route to enhance vaccine safety, inducing lower IL-10 levels and more interferon-γ-secreting cells (IFN-γ-SC), reduction of PRRSV shedding within the herd and decreasing the potential iatrogenic transfer of pathogens between pigs by the use of needles [19,20]. In addition, ID vaccination offers animal welfare benefits, eliminates accidental injuries in farm workers by needles, and reduces injection tissue damages caused by IM vaccine administration, which are quite prevalent in pigs in slaughterhouses [21].

A soluble glycosylated protein (molecular mass, 50–60 kDa), angiotensinogen is the only precursor of angiotensin (Ang) peptides as well as the sole known substrate for renin [22]. Angiotensinogen levels in blood circulation are relatively abundant (10^−5^ g or 10 μg/mL; 200 nM), especially compared with Ang II or Ang-(1–7), and for this reason, a minimal amount (<0.1 mL) of serum or plasma is needed to measure angiotensinogen [22]. An ELISA method for angiotensinogen detection and quantification has been developed by Kobori and colleagues (2008) [23] and is available commercially and comprises antibodies directed against two distinct antigenic regions for the capture and detection of angiotensinogen. Ang II is a major factor of the renin-angiotensin system (RAS) for the regulation of blood pressure and cardiovascular homeostasis and is considered a systemic hormone, deriving from an enzymatic cascade [24]. Renin is firstly released from the juxtaglomerular cells of the kidneys into the blood circulation, where it proteolytically causes angiotensinogen to create the decapeptide Ang I. Ang I can be then cleaved by angiotensin-converting enzyme (ACE) and produces the octapeptide Ang II within the pulmonary circulation [25,26,27]. Apart from the circulating RAS, Ang II is produced in many other tissues and organs (heart, vasculature, kidney and brain), in the presence of angiotensinogen, renin and ACE, a fact that implies the paracrine and intracrine effects of Ang II [25,26,27,28,29].

Ang II performs a crucial part in the inflammatory response, cell proliferation, migration and apoptosis [30,31,32,33]. It is known that Ang II promotes apoptosis in human and rat alveolar epithelial cells and obstructs apoptosis in pulmonary endothelial cells during acute lung injury [34,35]. Many studies reported that PRRSV causes apoptosis, necrosis and necrosis-like-apoptosis in lungs and lymphoid organs leading to a detrimental effect on the immune system of the infected pig [36,37,38,39,40]. In addition, Ang II is a second important factor of the Fas ligand (FasL) system in the lungs [41], while Fas/FasL is involved in PRRSV-induced apoptosis [42,43]. However, apoptosis could be induced in uninfected bystander cells by increased surface expression of FasL on surrounding cells caused by PRRSV infection, and as a result, these cells become sensitive to Fas-mediated apoptosis [42]. 

There are scarce published findings on the role of Ang II after IM or ID vaccination of swine against PRRSV. The ACE promotes the conversion of Ang I into vasoconstrictor Ang II and can be found in many tissues including vascular tissue [44]. ACE can promote vascular smooth muscle growth, capillary density and consumption of oxygen, affecting both sympathetic and neuromuscular transmission and having a hypertrophic effect on skeletal muscle, improving contractile function [45,46,47,48,49].

The goal of this study was to evaluate the concentration of Ang II in the serum of piglets following immunization against PRRSV with an MLV vaccine, through the ID and IM route, compared to non-vaccinated piglets.

## 2. Materials and Methods

### 2.1. Ethics

All procedures were performed according to the ethical standards and after receiving approval from the Animal Use Ethics Committee of Veterinary Faculty University of Thessaly (approval code: 98/19.12.2019). 

### 2.2. Farm

The current trial was performed in a commercial farrow-to-finish pig farm of 150 sows (Topigs Norsvin hybrids of Large White × Landrace). The farm also maintained a grandparent nucleus of 10 sows in order to breed its own gilts. The herd followed practices of a 1-week batch production method and weaned piglets at the age of 28 days. The group allocation of weaned piglets was equal, according to body weight and sex, at flat-deck batteries with an automatic climate-control system. The sows were vaccinated in a routine program against PRRSV, PCV2, swine influenza, porcine parvovirus, Erysipelothrix rhusiopathiae, Clostridium perfringens and Escherichia coli. Gilts were vaccinated against PRRSV twice before artificial insemination (180th and 210th days of age), and the sows underwent a 6th–60th vaccination scheme, using a commercial PRRSV MLV vaccine (Porcilis*^®^* PRRS, MSD Animal Health, Kenilworth, NJ, USA). Piglets were vaccinated against Mycoplasma hyopneumoniae and PCV2 at 21 days of age. The antiparasitic program of sows included injectable ivermectin once before farrowing, while boars were injected twice a year. 

### 2.3. Health History and Pre-Trial Period 

The trial farm had a previous PRRSV history, which included several severe outbreaks during the last ten years. PRRSV infection was verified by real-time polymerase chain reaction (qRT-PCR) examination performed in serum samples of sows/gilts as well as weaned, growing and finishing pigs). During past PRRSV outbreaks, clinical signs observed consisted of reproductive losses (increased abortion and returns to estrus rate, elevated number of mummified and stillborn piglets) accompanied with severe respiratory disease in the weaning stage associated with significant economic impact. Laboratory examinations in blood samples of unvaccinated suckling piglets for PRRSV showed a significant reduction of maternal antibodies against PRRSV at 14 days of age.

One month before the onset of the study, blood samples were taken from gilts, pregnant and lactating sows (12 total blood samples per group) and from suckling, weaned, growing and finishing pigs (8 samples at ages of 2, 4, 7, 10, 13, 17 and 21 weeks). Samples were screened for PRRSV genome by RT-PCR and the isolated viral strain was characterized via complete ORF5 Sanger sequencing. Additionally, the samples were examined for PCV2 by qRT-PCR.

Finally, nasal swab samples were tested by PCR for the detection of common respiratory pathogens (*Actinobacillus pleuropneumoniae, Mycoplasma hyopneumoniae, Streptococcus suis, Pasteurella multocida* and *Haemophilus parasuis*).

### 2.4. Study Design

The trial included 104 healthy suckling piglets (2 weeks of age), derived from 9 litters. The piglets were divided into four groups (A–D, Table 1) of 13 piglets and two replicates of the experimentation were performed (2 replicates × 13 piglets × 4 groups/26 piglets per group).

Diseased piglets and piglets with very poor condition (e.g., significantly low weight) or congenital abnormalities (e.g., hernias) were excluded from the trial. All animals included in the trial were distinguished by unique numbered ear tags and were housed and raised, mixed under the regular management program of the farm. 

A commercial PRRSV MLV vaccine (Porcilis^®^ PRRS, MSD Animal Health) was used in the current trial. The vaccine is approved for administration both via the IM or the ID way in the neck area, as stated in the Summary of Product Characteristics (SPC). Per vaccine dose of 2 mL (IM) or 0.2 mL (ID) of reconstituted lyophilized vaccine, the attenuated PRRSV strain DV is contained at titers 104.0–106.3 tissue culture infective dose 50%. Diluvac Forte^®^ (MSD Animal Health) is the adjuvant of the vaccine (containing 75 mg/mL dl-α tocopheryl acetate) that was administered to the piglets of the control groups.

Piglets from groups A and B were administered, at the age of 2 weeks, with one dose of Porcilis^®^ PRRS, diluted in 0.2 mL (group A, ID) or 2 mL (group B, IM) of Diluvac Forte^®^. Piglets of groups C and D were administered with Diluvac Forte^®^ only, using one dose of the recommended volume of adjuvant (0.2 mL for group C, or 2 mL for group D). An IDAL (IntraDermal Application of Liquids, MSD Animal Health) device was utilized to perform needle-free ID injection of experimental groups A and C. Pigs of IM administration groups (B and D) were injected with an automatic syringe (standard fixed volume 2 mL) and a new, sterile needle (size approx. 0.9 × 13 mm) was used for each group.

### 2.5. Sampling/Examinations

**Figure 1:**
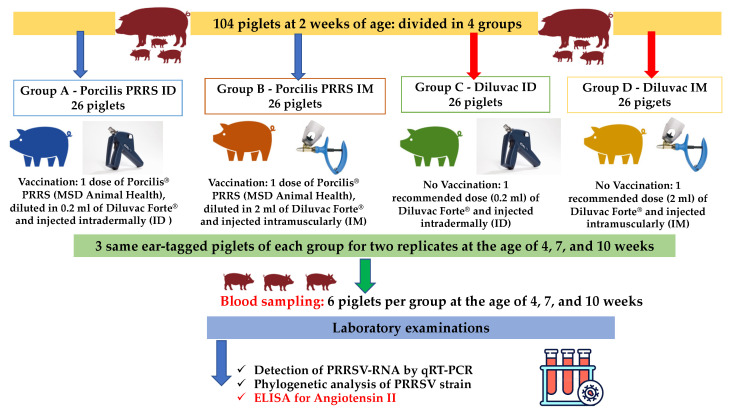
A flowchart of the trial design (experimental groups, vaccinations, sampling and laboratory examinations).

Blood samples were collected from each experimental group (three same ear-tagged piglets per group, for two replicates) at 4, 7 and 10 weeks of age, as shown in Figure 1. Blood serum samples were subjected to nucleic acid extraction with the use of the PureLink^®^ Viral RNA/DNA Mini Kit (Invitrogen, Carlsbad, CA, USA). Extracts were examined for the PRRSV genome, using an qRT-PCR assay [50]. Reactions were performed on a CFX96^®^ Real-Time System (Bio-Rad Laboratories, Hercules, CA, USA). Cycle threshold (Ct) values were used as viral load estimates. 

Serum samples were also tested by ELISA for the quantitative determination of Ang II levels (Pig angiotensin II, Ang II ELISA Kit; Catalogue number: CSB-E09370p, Cusabio, Wuhan, China. The method’s characteristics included a quantitative sandwich enzyme immunoassay technique, a sensitivity of 1.56 pg/mL. The assay used has high sensitivity and excellent specificity for detection of pig Ang Ⅱ. No significant cross-reactivity or interference between pig Ang II and analogues was noticed. The detection range was 6.25–400 pg/mL. The handling of blood samples was according to good laboratory practice.

In addition, nasal swab samples from the aforementioned six ear-tagged piglets per group were PCR tested for common respiratory pathogens (*Actinobacillus pleuropneumoniae, Mycoplasma hyopneumoniae, Streptococcus suis, Pasteurella multocida* and *Haemophilus parasuis*).

### 2.6. Statistical Analysis

The distribution of continuous data was tested for normality using the Kolmogorov–Smirnov test and they are shown as mean ± standard error and/or median and range (non-normal distribution). Comparisons were done applying the Kruskal–Wallis test while the Mann–Whitney U test was utilized for post hoc/multiple comparisons. Significance was set at 0.05. Statistical analysis was performed in IBM (IBM Corp., Armonk, NY, USA).

Correlation between Ang II blood serum levels and PRRSV viral load as measured by qRT-PCR was evaluated by the non-parametric Spearman’s correlation coefficient (ρ), using the commercial statistical software, MedCal 9.2 software (MedCalc Software, Mariakerke, Belgium). Significance was set at 0.05. The strength of the relationship was ranked as: ρ ≤ 0.35—weak correlations, 0.36 to 0.67—moderate correlations and 0.68 to 1.0—strong correlations [51].

## 3. Results

### 3.1. PCR Testing

PRRSV infection was verified before the onset of the trial by qRT-PCR in blood serum samples. Every sampled animal was qRT-PCR-positive for PRRSV. Analysis of the full-length ORF5 sequence of the wild-type PRRSV strain isolated in the trial farm at that time showed a 90.7% similarity (nucleotide sequence identity) with the DV strain (the data are available in Figure S1 of the Supplementary File). This strain is included in the commercial PRRSV MLV used in the current experiment. No positive samples for PCV2 were noticed.

The results of PCR testing for respiratory pathogens in nasal samples/during the pre-trial period were negative in all production stages that were sampled. Moreover, PCR screening for respiratory pathogens from experimental piglets revealed one positive unvaccinated pig at 7 weeks for *Streptococcus suis* and one positive unvaccinated pig at 10 weeks for *Streptococcus suis* and *Haemophilus parasuis*.

Findings of qRT-PCR in serum samples from pigs belonging to all groups at 4, 7 and 10 weeks of age are presented in Table 1. Specifically, pigs of all experimental groups were negative at the age of 4 weeks, and all pigs were positive at the age of 10 weeks, due to natural infection.

At the age of 7 weeks, all pigs of group A were negative, while 33.33% of pigs of group B and 83.33% of pigs of groups C and D were positive. However, the Ct value of qRT-PCR seems to be lower in group A compared to that of group B at the age of 10 weeks.

### 3.2. Angiotensin II Results

The results for Ang II levels are presented in Table 2. No significant differences were noticed in the same group over time. However, significant differences among groups were observed only at the age of 7 weeks (A vs. D, *p* = 0.004, B vs. D, *p* = 0.037, C vs. D, *p* = 0.006).

### 3.3. Correlation between PRRSV Viral Load and Ang II

Νο significant correlation was noticed either in the total number of animals at both time points or separately in each time point.

## 4. Discussion

Vaccination is one of the most common preventive tools applied in the swine industry. Nowadays, various vaccinations are often applied until finishing age. Even if IM vaccination is the most applied method in pigs, negative consequences (e.g., pain and necrosis) have been reported [52]. The needle-free ID vaccination is a less invasive and less painful alternative to IM vaccination [52,53,54,55]. The skin, due to its high density of immunocompetent cells, could be an alternative target tissue for vaccination [56]. The pro-inflammatory activity of Ang II is also mediated by activation of dendritic cells, highly specialized antigen-presenting cells responsible for immune response and inflammation defense. However, no data are published regarding to the role of Ang II after IM or ID vaccination against PRRSV in swine. The ACE promotes the conversion of Ang I to vasoconstrictor Ang II and is present in many tissues [44]. Ang II plays a key role in inflammation, through oxidative stress and production of adhesion molecules and cytokines [32]. In our study, a significant difference of mean Ang II concentration between ID and IM in piglets at 7 weeks of age. The increased mean Ang II concentration in IM groups could be possible correlated with the increased inflammation. The mean Ang II concentration in IM groups was decreased later (at 10 weeks of age), maybe due to recovery of piglets from inflammation.

In our study, at 7 weeks, only group A (ID PRRSV vaccinated) was qRT-PCR negative and had lower Ang II levels in comparison to group B (IM PRRSV vaccinated). In particular, at the age of 7 weeks, all animals of group A were qRT-PCR negative, while 33.33% of animals belonging to group B and 83.33% belonging to groups C and D were qRT-PCR positive. This finding provides evidence that ID vaccination induces less tissue damage and maybe affect the induction of sufficient protection. The diminished tissue damage is probably connected with lesser pain and distress. Behavioral alterations have been commonly utilized to assess pain and distress linked to injections in farm animals. A previous study, using the same commercial vaccine, demonstrated that ID-vaccinated suckling piglets exhibited more active behavior and suckling activity than piglets vaccinated through the IM route, 24 h after vaccination [57]. The needle-free ID vaccination method averts the acute phase response and muscle tissue damage related to IM injections [52]. Thus, ID vaccination offers important welfare benefits for the modern swine industry, as a less painful and less invasive alternative vaccine administration method when compared to IM injections [52].

Previous studies reported the regulatory role of PRRSV in induced apoptosis that are crucial to PRRSV pathogenesis [40,58,59,60]. Ang II, the key player of the renin-angiotensin system, has a crucial role in the inflammatory response, cell proliferation, migration and apoptosis [30,31,33]. In our previous study on ID and IM vaccination of piglets against PRRSV with the same vaccine, increased serum Fas levels were noticed in unvaccinated piglets, hinting apoptotic suppression when compared to vaccinated piglets. Moreover, higher sFAS levels were noticed the age of tested pigs increased, probably due to persistent PRRSV infection [61]. Ang II is a second important modulator of the FasL system in the lungs [41], while Fas/FasL is involved in PRRSV-induced apoptosis [42,43]. In our study, no significant differences in Ang II levels were noticed in the same group over time, while significant differences among groups were observed only at the age of 7 weeks. Due to Ang implication in the inflammation process, the absence of significant difference in Ang II levels in the same group could be attributed to the mild alteration of inflammation evidenced in all groups throughout the whole study period [32]. Moreover, no correlation between PRRSV viral load and Ang II was noticed in our study. Our results do not indubitably explain the role of Ang II in PRRSV-induced apoptosis. Previous studies, including comparison ID and IM MLV vaccination against PRRSV, reported that ID vaccination effectively induces specific humoral and cellular immune responses at least the same or even superior to IM vaccination [10,11,62]. Our results could be useful for researchers studying the effect of ID vaccination in piglets. However, future studies, including increased number of experimental animals and samples, are required to explore further the potential role of Ang II in the PRRSV induced apoptosis and elicited an immune response from ID or IM vaccination.

In conclusion, the present study provides evidence that ID PRRSV vaccination induces diminished tissue damage, based on the lower levels of Ang II in the serum of ID vaccinated piglets.

## Figures and Tables

**Table 1:** Results of qRT-PCR in blood serum samples of the sampled animals at ages of 4, 7 and 10 weeks.

Groups	Blood Sampling/Age		
	4 Weeks	7 Weeks	10 Weeks
	Number of PRRSV Positive Samples/Total Samples Average Ct Value (Min–Max)		
Group A	0/6	0/6	6/6
(Porcilis PRRS ID)	N/A	N/A	33.3 (30.5–36.5)
Group B	0/6	2/6	6/6
(Porcilis PRRS IM)	N/A	35.2 (32.3–38)	34.6 (29.5–39.7)
Group C	0/6	5/6	6/6
(Diluvac ID)	N/A	34.7 (25.5–40.8)	29.4 (25.4–32.9)
Group D	0/6	5/6	6/6
(Diluvac IM)	N/A	29.2 (24.2–32.2)	34.4 (25.6–39.0)

N/A: not applicable.

**Table 2:** Mean, standard error (SE), median and interquartile range (IQR) of angiotensin serum concentrations in all groups and time points.

Group	Time (Weeks)	Mean Angiotensin Concentration (×10^2^ pg/mL)	SE	Median	IQR
Group A (Porcilis PRRS ID)	4	0.25	0.25	0.00	0.00–0.00
	7	0.26 ^a^	0.21	0.06	0.00–0.14
	10	0.17	0.13	0.00	0.00–0.17
Group B (Porcilis PRRS IM)	4	2.97	1.86	0.90	0.09–3.63
	7	3.44 ^b^	2.51	1.05	0.28–2.31
	10	3.20	1.80	0.65	0.20–6.63
Group C (Diluvac ID)	4	1.06	0.37	0.83	0.62–1.32
	7	0.56 ^a^	0.42	0.08	0.03–0.45
	10	1.41	0.71	0.78	0.20–2.03
Group D (Diluvac IM))	4	4.13	2.30	1.14	0.17–7.52
	7	17.63 ^c^	7.51	11.37	4.73–25.14
	10	2.93	2.05	0.19	0.01–3.64

^a,b,c^ Different superscript letters denote significant differences (*p* < 0.05).

## Data Availability

The data presented in this study are available on request from the corresponding author. The data are not publicly available due to further processing for another publication.

## Supplementary Materials

The following supporting information can be downloaded at: https://www.mdpi.com/article/10.3390/vetsci9090496/s1, Figure S1. Comparison of ORF 5 sequences between the DV vaccine strain and the wild type virus circulating in the farm.

## References

[1] Papatsiros V.G., Perez-Marin C.C. (2012). Porcine Herd Health Management Practices for the Control of PRRSV Infection. A Bird’s-Eye View of Veterinary Medicine.

[2] Zimmerman J.J., Benfield D.A., Dee S.A., Murtaugh M.P., Stadejek T., Stevenson G.W., Torremorell M. (2012). Porcine Reproductive and Respiratory Syndrome. Diseases of Swine.

[3] Papatsiros V.G., Alexopoulos C., Kritas S.K., Koptopoulos G., Nauwynck H.J., Pensaert M.B., Kyriakis S.C. (2006). Long-term administration of a commercial porcine reproductive and respiratory syndrome virus (PRRSV)-inactivated vaccine in PRRSV-endemically infected sows. J. Vet. Med. B.

[4] Linhares D.C., Johnson C., Morrison R.B. (2016). Correction: Economic Analysis of Vaccination Strategies for PRRS Control. PLoS ONE.

[5] Charerntantanakul W. (2012). Porcine reproductive and respiratory syndrome virus vaccines: Immunogenicity, efficacy, and safety aspects. World J. Virol..

[6] Nan Y., Wu C., Gu G., Sun W., Zhang Y.J., Zhou E.M. (2017). Improved Vaccine against PRRSV: Current Progress and Future Perspective. Front. Microbiol..

[7] Romani N., Flacher V., Tripp C.H., Sparber F., Ebner S., Stoitzner P. (2012). Targeting skin dendritic cells to improve intradermal vaccination. Curr. Top. Microbiol. Immunol..

[8] Teunissen M., Haniffa M., Collin M. (2012). Insight, into the immunobiology of human skin and functional specialization of skin dendritic cell subsets to innovate intradermal vaccination design. Curr. Top. Microbiol. Immunol..

[9] Mikulska-Skupien E., Szweda W., Procajlo Z., Platt-Samoraj A. (2004). Indices of nonspecific cellular immune response in pigs after intradermal vaccination with deleted Aujeszky’s disease vaccine and after experimental infection. Bull. Vet. Inst. Pulawy.

[10] Martelli P., Cordioli P., Alborali L.G., Gozio S., De Angelis E., Ferrari L., Lombardi G., Borghetti P. (2007). Protection and immune response in pigs intradermally vaccinated against porcine reproductive and respiratory syndrome (PRRS) and subsequently exposed to a heterologous European (*Italian cluster*) field strain. Vaccine.

[11] Martelli P., Gozio S., Ferrari L., Rosina S., De Angelis E., Quintavalla C., Bottarelli E., Borghetti P. (2009). Efficacy of a modified live porcine reproductive and respiratory syndrome virus (PRRSV) vaccine in pigs naturally exposed to a heterologous European (*Italian cluster*) field strain: Clinical protection and cell-mediated immunity. Vaccine.

[12] Baker S.R., Mondaca E., Polson D., Dee S.A. (2012). Evaluation of a needle-free injection device to prevent hematogenous transmission of porcine reproductive and respiratory syndrome virus. J. Swine Health Prod..

[13] Stadler J., Naderer L., Beffort L., Ritzmann M., Emrich D., Hermanns W., Fiebig K., Saalmüller A., Gerner W., Glatthaar-Saalmüller B. (2018). Safety and immune responses after intradermal application of Porcilis PRRS in either the neck or the perianal region. PLoS ONE.

[14] Madapong A., Saeng-Chuto K., Chaikhumwang P., Tantituvanont A., Saardrak K., Pedrazuela Sanz R., Miranda Alvarez J., Nilubol D. (2020). Immune response and protective efficacy of intramuscular and intradermal vaccination with porcine repro- ductive and respiratory syndrome virus 1 (PRRSV-1) modified live vaccine against highly pathogenic PRRSV-2 (HP-PRRSV-2) challenge, either alone or in combination with of PRRSV-1. Vet. Microbiol..

[15] Sno M., Cox E., Holtslag H., Nell T., Pel S., Segers R., Fachinger V., Witvliet M. (2016). Efficacy and safety of a new intradermal PCV2 vaccine in pigs. Trials Vaccinol..

[16] Ellegaard B., Korsgarrd J., Nielsen G.B. (2021). Comparison of intradermal and intramuscular porcine circovirus type 2 vaccination methods concerning labor, production parameters, and antimicrobial treatments: A randomized field study in a Danish finishing herd. J. Swine Health Prod..

[17] Tassis P.D., Papatsiros V.G., Nell T., Maes D., Alexopoulos C., Kyriakis S.C., Tzika E.D. (2012). Clinical evaluation of intradermal vaccination against porcine enzootic pneumonia (*Mycoplasma hyopneumoniae*). Vet. Rec..

[18] Lee S.I., Jeong C.G., Ul Salam Mattoo S., Nazki S., Prasad Aganja R., Kim S.C., Khatun A., Oh Y., Noh S.H., Lee S.M. (2021). Protective immunity induced by concurrent intradermal injection of porcine circovirus type 2 and Mycoplasma hyopneumoniae inactivated vaccines in pigs. Vaccine.

[19] Pileri E., Mateu E. (2016). Review on the transmission porcine reproductive and respiratory syndrome virus between pigs and farms and impact on vaccination. Vet. Res..

[20] Madapong A., Saeng-Chuto K., Tantituvanont A., Nilubol D. (2021). Safety of PRRSV-2 MLV vaccines administrated via the intramuscular or intradermal route and evaluation of PRRSV transmission upon needle-free and needle delivery. Sci. Rep..

[21] Cannon J.E. (1995). Pork quality audit: A review of the factors influencing pork quality 1. J. Muscle Foods.

[22] Chappell M.C. (2016). Biochemical evaluation of the renin-angiotensin system: The good, bad, and absolute?. Am. J. Physiol. Heart Circ. Physiol..

[23] Kobori H., Katsurada A., Miyata K., Ohashi N., Satou R., Saito T., Hagiwara Y., Miyashita K., Navar L.G. (2008). Determination of plasma and urinary angiotensinogen levels in rodents by newly developed ELISA. Am. J. Physiol. Renal Physiol..

[24] Lavoie J.L., Sigmund C.D. (2003). Minireview: Overview of the renin-angiotensin system-an endocrine and paracrine system. Endocrinology.

[25] Campbell D.J. (1987). Circulating and tissue angiotensin systems. J. Clin. Investig..

[26] Johnston C.I. (1992). Franz Volhard Lecture. Renin-angiotensin system: A dual tissue and hormonal system for cardiovascular control. J. Hypertens. Suppl. Off. J. Int. Soc. Hypertens..

[27] Wen H., Gwathmey J.K., Xie L.H. (2012). Oxidative stress-mediated effects of angiotensin II in the cardiovascular system. World J. Hypertens..

[28] Phillips M.I., Speakman E.A., Kimura B. (1993). Levels of angiotensin and molecular biology of the tissue renin angiotensin systems. Regul. Pept..

[29] Vinson G.P., Ho M.M., Puddefoot J.R. (1995). The distribution of angiotensin II type 1 receptors, and the tissue renin-angiotensin systems. Mol. Med. Today.

[30] Hernández J.S., Barreto-Torres G., Kuznetsov A.V., Khuchua Z., Javadov S. (2014). Crosstalk between AMPK activation and angiotensin II-induced hypertrophy in cardiomyocytes: The role of mitochondria. J. Cell Mol. Med..

[31] Richardson M.A., Gupta A., O’Brien L.A., Berg D.T., Gerlitz B., Syed S., Sharma G.R., Cramer M.S., Heuer J.G., Galbreath E.J. (2008). Treatment of sepsis-induced acquired protein C deficiency reverses Angiotensin-converting enzyme-2 inhibition and decreases pulmonary inflammatory response. J. Pharmacol. Exp. Ther..

[32] Marchesi C., Paradis P., Schiffrin E.L. (2008). Role of the renin-angiotensin system in vascular inflammation. Trends Pharmacol. Sci..

[33] Hagiwara S., Iwasaka H., Matumoto S., Hidaka S., Noguchi T. (2009). Effects of an angiotensin-converting enzyme inhibitor on the inflammatory response in in vivo and in vitro models. Crit. Care Med..

[34] Wang R., Zagariya A., Ibarra-Sunga O., Gidea C., Ang E., Deshmukh S., Chaudhary G., Baraboutis J., Filippatos G., Uhal B.D. (1999). Angiotensin II induces apoptosis in human and rat alveolar epithelial cells. Am. J. Physiol..

[35] Ji Y., Gao F., Sun B., Hao J., Liu Z. (2015). Angiotensin-converting enzyme 2 inhibits apoptosis of pulmonary endothelial cells during acute lung injury through suppressing SMAD2 phosphorylation. Cell Physiol. Biochem..

[36] Gómez-Laguna J., Salguero F.J., Pallarés F.J., Carrasco L. (2013). Immunopathogenesis of porcine reproductive and respiratory syndrome in the respiratory tract of pigs. Vet. J..

[37] Morgan S.B., Frossard J.P., Pallares F.J., Gough J., Stadejek T., Graham S.P., Steinbach F., Drew T.W., Salguero F.J. (2016). Pathology and virus distribution in the lung and lymphoid tissues of pigs experimentally inoculated with three distinct Type 1 PRRS virus isolates of varying pathogenicity. Transbound. Emerg. Dis..

[38] Costers S., Lefebvre D.J., Delputte P.L., Nauwynck H.J. (2008). Porcine reproductive and respiratory syndrome virus modulates apoptosis during replication in alveolar macrophages. Arch. Virol..

[39] Rodríguez-Gómez I.M., Barranco I., Amarilla S.P., García-Nicolás O., Salguero F.J., Carrasco L., Gómez-Laguna J. (2014). Activation of extrinsic-and Daxx-mediated pathways in lymphoid tissue of PRRSV-infected pigs. Vet. Microbiol..

[40] Labarque G., Van Gucht S., Nauwynck H., Van Reeth K., Pensaert M. (2003). Apoptosis in the lungs of pigs infected with porcine reproductive and respiratory syndrome virus and associations with the production of apoptogenic cytokines. Vet. Res..

[41] Wang R., Zagariya A., Ang E., Ibarra-Sunga O., Uhal B.D. (1999). Fas-induced apoptosis of alveolar epithelial cells requires ANG II generation and receptor interaction. Am. J. Physiol..

[42] Lee S.M., Kleiboeker S.B. (2007). Porcine reproductive and respiratory syndrome virus induces apoptosis through a mitochondria-mediated pathway. Virology.

[43] Chang H.W., Jeng C.R., Lin C.M., Liu J.J., Chang C.C., Tsai Y.C., Chia M.Y., Pang V.F. (2007). The involvement of Fas/FasL interaction in porcine circovirus type 2 and porcine reproductive and respiratory syndrome virus co-inoculation-associated lymphocyte apoptosis in vitro. Vet Microbiol..

[44] Dezsö B., Jacobsen J., Poulsen K. (1989). Evidence for the presence of angiotensins in normal, unstimulated alveolar macrophages and monocytes. J. Hypertens..

[45] Jones A., Woods D.R. (2003). Skeletal muscle RAS and exercise performance. Int. J. Biochem. Cell Biol..

[46] Dietze G.J., Henriksen E.J. (2008). Angiotensin-converting enzyme in skeletal muscle: Sentinel of blood pressure control and glucose homeostasis. J. Renin Angiotensin Aldosterone Syst..

[47] Alves C.R., Alves G.B., Pereira A.C., Trombetta I.C., Dias R.G., Mota G.F., Fernandes T., Krieger J.E., Negrão C.E., Oliveira E.M. (2013). Vascular reactivity and ACE activity response to exercise training are modulated by the +9/−9 bradykinin B₂ receptor gene functional polymorphism. Physiol. Genomics.

[48] Vaughan D., Huber-Abel F.A., Graber F., Hoppeler H., Fluck M. (2013). The angiotensin converting enzyme insertion/deletion polymorphism alters the response of muscle energy supply lines to exercise. Eur. J. Appl. Physiol..

[49] Vaughan D., Brogioli M., Maier T., White A., Waldron S., Rittweger J., Toigo M., Wettstein J., Laczko E., Flück M. (2016). The angiotensin-converting enzyme insertion/deletion polymorphism modifies exercise-induced muscle metabolism. PLoS ONE.

[50] Lurchachaiwong W., Payungporn S., Srisatidnarakul U., Mungkundar C., Theamboonlers A., Poovorawan Y. (2008). Rapid detection and strain identification of porcine reproductive and respiratory syndrome virus (PRRSV) by real-time RT-PCR. Lett. Appl. Microbiol..

[51] Taylor R. (1990). Interpretation of the Correlation Coefficient: A Basic Review. J. Diagn. Med. Sonogr..

[52] Temple D., Jiménez M., Escribano D., Martín-Valls G., Díaz I., Manteca X. (2020). Welfare Benefits of Intradermal Vaccination of Piglets. Animals.

[53] Mitragotri S. (2005). Immunization without needles. Nat. Rev. Immunol..

[54] Mitragotri S. (2006). Current status and future prospects of needle-free liquid jet injectors. Nat. Rev. Drug Discov..

[55] Temple D., Escribano D., Jiménez M., Mainau E., Cerón J.J., Manteca X. (2017). Effect of the needle-free “intra dermal application of liquids” vaccination on the welfare of pregnant sows. Porc. Health Manag..

[56] Bangert C., Brunner P.M., Stingl G. (2011). Immune functions of the skin. Clin. Dermatol..

[57] Göller M., Knöppel H.P., Fiebig K., Kemper N. Intradermal vaccine application: Effects on suckling behaviour. Proceedings of the 24th International Pig Veterinary Society Congress.

[58] Sur J.H., Doster A.R., Osorio F.A. (1998). Apoptosis induced in vivo during acute infection by porcine reproductive and respiratory syndrome virus. Vet. Pathol..

[59] He Y., Wang G., Liu Y., Shi W., Han Z., Wu J., Jiang C., Wang S., Hu S., Wen H. (2012). Characterization of thymus atrophy in piglets infected with highly pathogenic porcine reproductive and respiratory syndrome virus. Vet. Microbiol..

[60] Karniychuk U.U., Saha D., Geldhof M., Vanhee M., Cornillie P., Van den Broeck W., Nauwynck H.J. (2011). Porcine reproductive and respiratory syndrome virus (PRRSV) causes apoptosis during its replication in fetal implantation sites. Microb. Pathog..

[61] Maragkakis G., Korou L.M., Chaintoutis S.C., Christodoulopoulos G., Dovas C.I., Perrea D., Athanasiou L.V., Konstantopoulos P., Maes D., Papatsiros V.G. (2022). Investigation of Fas (APO-1)-Related Apoptosis in Piglets Intradermally or Intramuscularly Vaccinated with a Commercial PRRSV MLV. Viral Immunol..

[62] Ferrari L., Martelli P., Saleri R., De Angelis E., Cavalli V., Bresaola M., Benetti M., Borghetti P. (2013). Lymphocyte activation as cytokine gene expression and secretion is related to the porcine reproductive and respiratory syndrome virus (PRRSV) isolate after in vitro homologous and heterologous recall of peripheral blood mononuclear cells (PBMC) from pigs vaccinated and exposed to natural infection. Vet. Immunol. Immunopathol..

