# Supplementary material for: Angiotensin II Blood Serum Levels in Piglets, after Intra-Dermal or Intra-Muscular Vaccination against PRRSV
Source: Vet Sci. 2022 Sep 11;9(9):496. doi: 10.3390/vetsci9090496 (PMC9503611; doi:10.3390/vetsci9090496)
Supplement: Supplementary file 1 [file vetsci-09-00496-s001.zip › vetsci-1825110-supplementary.pdf]

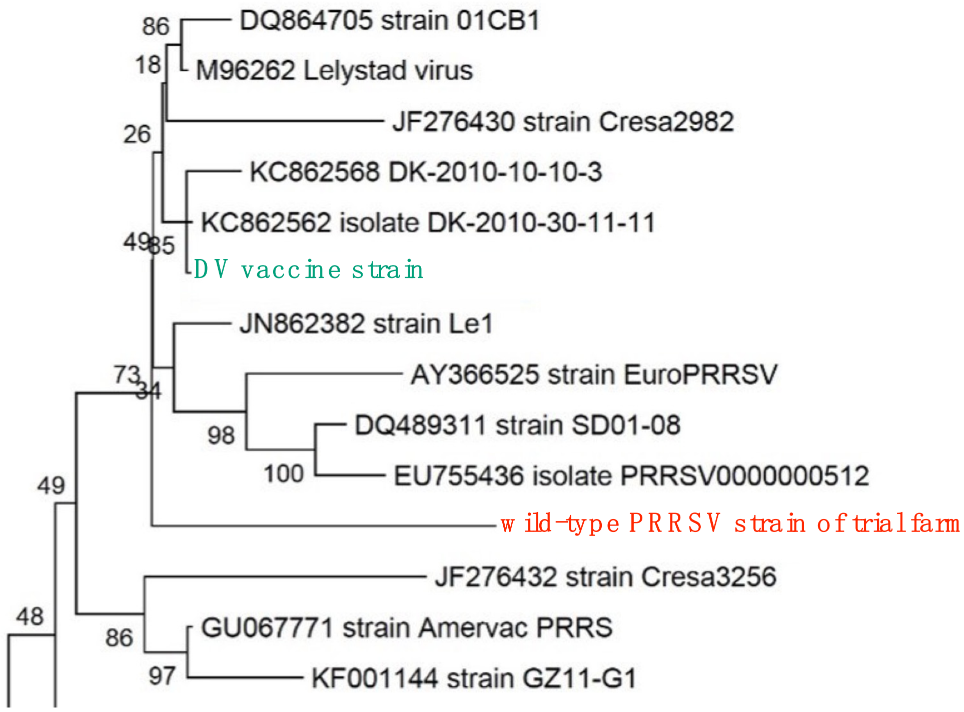

Figure S1. Comparison of ORF 5 sequences between the DV vaccine strain and the wild-type PRRSV strain of the trial farm: 90.7% sequence identity
